# Fissure of the Anus

**Published:** 1883-12

**Authors:** Thos. Hayd


### Selections.

### Fissure of the Anus.

The value and efficacy of iodoform in fissure of the anus will bring this remedy into general use in the treatment of this painful and heretofore incurable lesion, without operation by the knife or forcible rupture of the sphincter-ani muscle.

As in cases involving the greatest danger, so with fissure of the anus, if the trouble can be cured by simple means, without suffering to the patient, and in reasonably due time, the operation of cutting or forcible rupture is not justifiable, and both these means of radical cure must give way to the more simple, if such may exist. With the experience I have had in the use of the local application of iodoform in cases of fissure of the anus, I am encouraged to bring the value of this remedy to the notice of the profession in these cases. In their treatment with this remedy, the alvine evacuations should always be maintained in a soft condition, the bowels should never be allowed to become constipated or relaxed; the anus, and parts involved by the fissure, should be kept constantly clean and free from deposit and dry incrustations; and, with one or two evacuations a day, the case may be speedily cured by the local use of iodoform. It may be dusted, in very fine powder, upon and into the fissured parts, or applied in the form of ointment or suppository. The application of the simple powders, if properly prepared, three or four times a day after each evacuation, and in the intervals, is often sufficient. In some cases, however, the undiluted powder—also thoroughly powdered—causes some pain. In such the iodoform may be mixed with powdered gum acacia, if a powder be preferred, or may be made into an ointment with vaseline, or suppository with the oil of theobroma. Balsam of Peru, carbolic acid and oil of peppermint, will moderate the intensity of the iodoform odor; but this can hardly be requisite for application in this situation. The application of the remedy may be followed by a little smarting, but soon after its use the sensibility of the parts becomes benumbed, and even defecation may go on without consciousness, so far as concerns the development of pain during or after the process. That this remedy, applied as above directed and indicated will cause complete unconsciousness of the act of defecation, I doubt; I have never witnessed such result in any case that has come under my notice, and still the benumbing influence of the remedy is decidedly potent. As in applications to the conjunctival surfaces of the eyelids, the first and most important factor in the successful and painless use of the remedy consists in the proper preparation of the powder. It should be made very fine, and not the smallest crystal be allowed to remain unpowdered. The neglect of this precaution when applied to the eye has caused the most painful inflammation of the ocular and palpebral conjunctiva, and, applied thus imperfectly powdered to the anus, would likewise cause intense suffering, and, as in eye-practice, would be abandoned and declared to be dangerous and valueless, if intelligence did not bring relief.—*Thos. Hayd, M. D., in Medical and Surgical Reporter.*

